# Reassortant Highly Pathogenic H5N6 Avian Influenza Virus Containing Low Pathogenic Viral Genes in a Local Live Poultry Market, Vietnam

**DOI:** 10.1007/s00284-021-02661-z

**Published:** 2021-09-21

**Authors:** Tran Bac Le, Van Phan Le, Ji-Eun Lee, Jung-Ah Kang, Thi Bich Ngoc Trinh, Hyeok Won Lee, Dae Gwin Jeong, Sun-Woo Yoon

**Affiliations:** 1 Bio-Nanotechnologytechnology Research Center, Korea Research Institute of Bioscience and Biotechnology, Daejeon, 34141 South Korea; 2 Bio-Analytical Science Division, University of Science and Technology, Daejeon, 34113 South Korea; 3 College of Veterinary Medicine, Vietnam National University of Agriculture, Hanoi, 100000 Vietnam; 4 Bio-Process Engineering Center, Korea Research Institute of Bioscience and Biotechnology, Daejeon, 34141 Korea

## Abstract

Sites of live poultry trade and marketing are hot spots for avian influenza virus (AIV) transmission. We conducted active surveillance at a local live poultry market (LPM) in northern Vietnamese provinces in December 2016. Feces samples from the market were collected and tested for AIV. A new reassorted AIV strain was isolated from female chickens, named A/chicken/Vietnam/AI-1606/2016 (H5N6), and was found to belong to group C of clade 2.3.4.4 H5N6 highly pathogenic (HP) AIVs. The neuraminidase gene belongs to the reassortant B type. The viral genome also contained polymerase basic 2 and polymerase acidic, which were most closely related to domestic-duck-origin low pathogenic AIVs in Japan (H3N8) and Mongolia (H4N6). The other six genes were most closely related to poultry-origin H5N6 HP AIVs in Vietnam and had over 97% sequence identity with human AIV isolate A/Guangzhou/39715/2014 (H5N6). The new reassorted AIV isolate A/chicken/Vietnam/AI-1606/2016 (H5N6) identified in this study exemplifies AIVs reassortment and evolution through contact among wild birds, poultry farms, and LPMs. Therefore, active surveillance of AIVs is necessary to prevent potential threats to human and animal health.

**Supplementary Information:**

The online version contains supplementary material available at 10.1007/s00284-021-02661-z.

## Introduction

Avian influenza viruses (AIVs) belong to the family *Orthomyxoviridae.* Among the AIV subtypes identified in wild aquatic birds based on two surface glycoproteins, hemagglutinin (HA1 to 16) and neuraminidase (NA1 to 9), H5Nx subtypes have been a major concern in the poultry industry and public health since the first highly pathogenic avian influenza virus (HPAIV) H5N1 subtype was detected in a goose in Guangdong (Gs/GD) province, China, 1996 [1, 2]. Based on their pathogenicity in chickens and the multi-basic cleavage site motif in the HA protein, H5Nx subtypes have both low pathogenic (LP) and HPAIV types [3]. Aquatic birds, being the major natural reservoir of AIVs, are subject to natural migration and factitious trading in live poultry markets (LPMs). Interaction among them through the intersection of wild birds and poultry sector, together with the specialized genome organization of AIVs, these factors have created numerous clades of H5 HPAIV and reassortant strains, which are variants differing in NA subtypes, such as H5N1, H5N2, H5N5, H5N6, and H5N8. These strains, along with variants originating from other internal genes, have caused outbreaks in poultry in over 80 countries worldwide, including China, Japan, South Korea, Laos, and Vietnam thus far [4].

In 2013, the first clade 2.3.4.4 H5N6 HPAIV was identified and was the result of a reassortant between the HPAIV H5N1 subtype and LPAIV H6N6, which circulate broadly in duck populations in southern and eastern China [5]. Reassortants with six internal genes such as PB2, PB1, PA, NP, M, and NS in H5N6 from the chicken H9N2 or H7N9 gene pool, have also been reported [6]. As of May 2020, 24 human cases of H5N6 HPAIV infection have occurred exclusively in China since 2014, and a mortality rate of ~66% (16/24) was reported by the World Health Organization (WHO) [7]. Occasionally, H5N6 HPAI viruses have also been detected in mammals such as pigs and domestic cats [8, 9]. Swine have been considered for intermediate transmission of influenza A viruses between birds and humans because they have both α2,6- and α2,3-linked sialic acid receptors [1].

In Vietnam, the first Gs/GD lineage H5N1 HPAIV was detected in 2001 [10], and numerous clades have been identified since then [11–14]. Clade 1 was the primary cause of the first wave of massive outbreaks in poultry during 2003–2005 and was subsequently replaced by other clades, such as clade 2.3.2 in 2005–2008 and 2.3.4 in 2007–2010 in the North and Central regions. Clade 1 and its descendants, including clade 1.1.1 and 1.1.2, continued to predominate in the South region. Clade 2.3.2.1 (a/b/c) replaced clade 2.3.4 in parts of the North and Central regions beginning in 2009, and clade 2.3.2.1c spread to the South region after 2012. Since 2014, clades 2.3.2.1c and 2.3.4.4 have become concurrently predominant in Vietnam.

LPMs are integral components of the poultry trade network in Vietnam. The behavior of poultry traders in Vietnamese markets is a potential risk factor for human and animal health, making it more likely for AIVs to overcome the species barrier [15]. Hence, enhanced surveillance activity and complete viral genetic analyses are necessary for virus tracing and maintenance of human and animal health. In this report, we isolated and genetically characterized a new reassortant clade 2.3.4.4 group C H5N6 HPAIV strain from female chicken feces collected from LPMs in northern Vietnamese provinces in December 2016. Our findings have extended our understanding of H5N6 HPAIV genetic diversity in poultry and how the trading network in Vietnam affects AIV evolution.

## Materials and Methods

During the winter between 2016 and 2017, feces sampling in poultry was carried out at local LPMs and small-scale poultry farms in Ha Nam province, Vietnam (approximately 60 km from Hanoi to the south). A total 353 of fecal, tracheal, and cloacal swab samples were collected from four local live bird markets and poultry farms and plated into transportation medium (Noble Biosciences, South Korea) and stored at −80°C. Samples were then tested for AIVs using a matrix (M) gene-specific real-time reverse transcription quantitative polymerase chain reaction (RT-qPCR) method according to the WHO guideline for animal influenza virus detection [16]. Continuously, the samples containing influenza A virus were examined for HPAIV and clade detection based on an RT-qPCR assay following a previous report [17]. Positive AIV samples were isolated by inoculating 10-day-old embryonated chicken eggs in a biosafety level two plus facility at the College of Veterinary Medicine, Vietnam National University of Agriculture, Hanoi, Vietnam. The host sex and species was determined by analyzing the mitochondrial cytochrome oxidase (COI) and chromo-domain helicase DNA binding (CHD) genes following a previous report [18, 19].

For complete viral genome analysis of the new isolate, viral RNA was prepared from the allantoic fluid from the first egg passage using a QIAamp Viral RNA Mini Kit (Qiagen, CA, USA) following the manufacturer’s protocol. Viral genome amplification was performed using the conventional RT-PCR method with PrimeScript™ First-Strand cDNA Synthesis Kit and Premix Taq™ (Takara, Japan) with universal primers previously described [20]. The DNA band for each target gene was excised from a 1% agarose gel and DNA was purified using the QIAquick Gel Extraction Kit (Qiagen, CA, USA). The purified DNA was then subjected to direct sequencing using an ABI3730XL DNA analyzer (Cosmo Genetech Service, South Korea). The sequences were assembled using CLC Sequence Viewer software version 6.7. Non-coding regions containing primer sequences were trimmed. The open reading frames of eight genes were submitted to GenBank under accession number MT634255-MT634262. A BLAST search on the GenBank database was used to determine the closest related strains to the newly isolated virus.

Phylogenetic analysis based on each gene sequence was conducted using Molecular Evolutionary Genetics Analysis Version 7.0 software (MEGA 7.0). The evolutionary distances were computed using the Maximum Composite Likelihood method with 1000 replicates. Input nucleotide sequences included both the new isolate and reference sequences from the open access resources for the GenBank database of influenza virus. The length of eight segments containing the PB2, PB1, PA, HA, NP, NA, M, and NS genes used for phylogenetic analysis were 2280, 2274, 2151, 1704, 1497, 1380, 982, and 823 nucleotides, respectively.

## Results

**Fig. 1:**
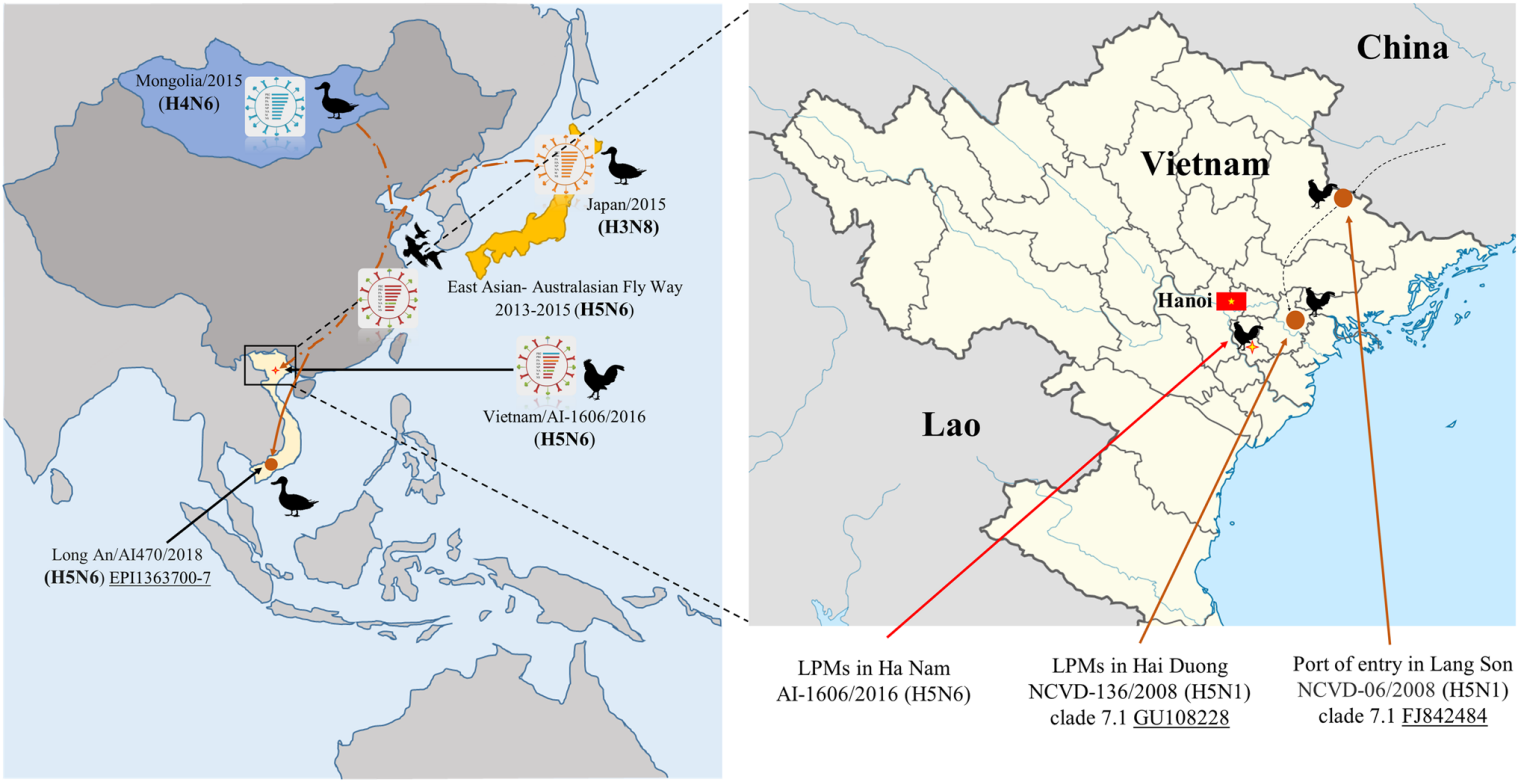
Location of putative origins of genomic compositions of the new reassortant A/chicken/Vietnam/AI-1606/2016 (H5N6). The brown circles were isolates in previous reports and red arrows were reassortant A/chicken/Vietnam/AI-1606/2016 isolate in this report

Among the eight confirmed positive samples of influenza A virus by M-gene-specific RT-qPCR, we identified three of H5N1 and four of H9N2 avian influenza viruses (Supplementary Table 1). Interestingly, a fecal sample collected from a local LPM on December 30th, 2016 was suspected to be positive for HPAIV. In the first egg passage, the isolate showed signs of HPAI virus infection, such as death of the embryos and allantoic fluid in red at 36 h post-inoculation. The new reassortant strain of clade 2.3.4.4c H5N6 HPAIV was identified and named A/Chicken/Vietnam/AI-1606/2016 (abbreviated as A/AI-1606/16). The geographical location of the A/AI-1606/16 isolate was shown in Fig. 1. A female chicken (*Gallus gallus*) was the host of A/AI-1606/16 followed to COI and CHD partial genes analysis (Supplementary Fig. 1).

**Fig. 2:**
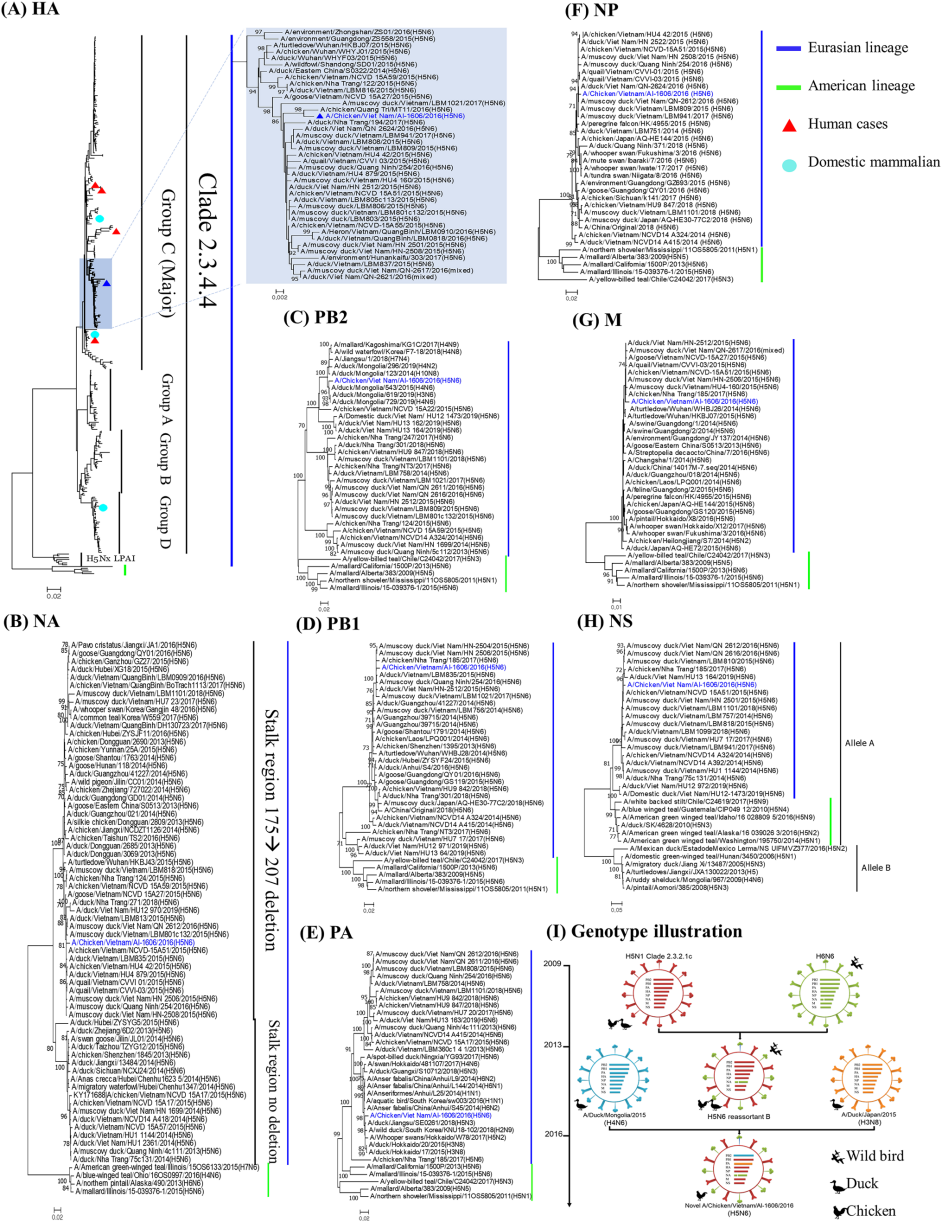
Phylogenetic and genotype illustration analysis of A/chicken/Vietnam/AI-1606/2016 (H5N6) virus. Phylogenetic tree based on nucleotide sequences of eight genes (**A**–**H**) were conducted using Molecular Evolutionary Genetics Analysis Version 7.0. The evolutionary distances were computed using the Maximum Composite Likelihood method with 1000 replicates. The input nucleotide sequences included both new isolate and reference sequences obtained from the influenza virus resource at the National Center for Biotechnology Information (NCBI). The statistic values greater than 70% a measure of reliability from a bootstrap (*n* = 1000) iterations were showed. Genotype illustration (I) of the new reassortant A/chicken/Vietnam/AI-1606/2016 (H5N6)

Based on the genetic analysis, suggested that the A/AI-1606/16 isolate was closely related to domestic-poultry-origin AIVs. The six segments of PB1, HA, NP, NA, M, and NS genes were most closely related to the H5N6 HPAIV virus that circulated among domestic poultry in Vietnam between 2015 and 2016. The sequence similarity was greater than 99%. However, the PB2 gene was most closely related to A/duck/Mongolia/543/2015(H4N6), with 98.82% identity; and the PA gene was most closely related to A/duck/Hokkaido/20/2015 (H3N8), with 98.84% identity. The closest similarities of the eight gene segments of the A/AI-1606/16 virus are listed in Table 1, and Fig. 2I shows how the virus may have been generated.

**Table 1:** Closest related viruses and identification of amino acids deduction of A/chicken/Vietnam/AI-1606/2016 (H5N6) isolate involved in binding to human-type influenza receptor, enhancing antiviral drug resistance, and causing pathogenesis in mammals

Top BLAST H5N6 HPAI strains	Viral protein*			Molecular and phenotypic impact of amino acid [reference]
A/chicken/Quang Tri/MT11/2016 (H5N6)	HA	PLRERRRKR↓GLF	Cleavage site	Expanded viral tropism; increased virulence in mice [8]
		107Y	H→Y	H5 transmissibility in ferrets [8]
		137A	S→A	Increased α2,6-SA recognition [21]
		160A	T→A	H5 transmissibility in ferrets [8]
A/chicken/Vietnam/NCVD-15A51/2015 (H5N6)	NA	59→69 deletion	Stalk region	Increased virulence in mice [8]
		274H	H→Y	Oseltamivir resistance [22]
		294 N	N→S	Oseltamivir resistance [22]
A/duck/Mongolia/543/2015 (H4N6)	PB2	89 V	L→V	Increased pathogenicity in mice [21]
		309D	G→D	Increased virulence and replication in mice [21]
		339 K	T→K	Increased virulence and replication in mice [21]
		477G	R→G	Increased virulence and replication in mice [21]
		495 V	I→V	Increased virulence and replication in mice [21]
A/muscovy duck/Viet Nam/HN-2504/2015 (H5N6)	PB1	99H	H→Y	H5 transmissibility in ferrets [8]
		368I	I→V	H5 transmissibility in ferrets [8]
A/duck/Hokkaido/20/2015 (H3N8)	PA	97 T	T→I	Enhanced polymerase activity; increased virulence [8]
	PA-X	195R	R→K	Increased replication and transmission in ferrets [23]
A/muscovy duck/Viet Nam/QN-2612/2016 (H5N6)	NP	286A	A→V	Attenuated the virulence in mice [24]
		319 N	N→K	Enhanced replication efficiency [25]
		437 T	T→M	Attenuated the virulence in mice [24]
A/duck/Vietnam/HU4-879/2015 (H5N6)	M1	30D	N→D	Increased virulence in mice [8]
		215A	T→A	Increased virulence in mice [8]
	M2	31S	S→N	Amantadine resistance [26]
A/duck/Vietnam/HU4-879/2015 (H5N6)	NS1	42S	P→S	Increased virulence in mice [8]
		92E	D→E	Increased virulence in mice and pigs [21]
		80→84 deletion ESEV	C-terminal PDZ-motif	Increased viral virulence in chicken and mice [27] Increased virulence in mice [21]

*Amino acid position, *A* alanine; *D* aspartic acid; *E* glutamic acid; *G* glycine; *H* histidine; *I* Isoleucine; *K* lysine; *L* leucine; *M* methionine; *N* asparagine; *P* proline; *Q* glutamine; *R* arginine; *S* serine; *T* threonine, *V* valine; *Y* tyrosine

In the phylogenetic tree analysis, the eight genes of the A/AI-1606/16 virus belonged to Eurasian lineages (Fig. 2A–H). The HA gene fell into group C of clade 2.3.4.4, the NA gene grouped into reassortant B type, and the PB2 and PA genes associated with LPAIVs; domestic ducks in Mongolia (H4N6) and Japan (H3N8), respectively. The NS gene belonged to allele A.

The predicted amino acid sequence of the A/AI-1606/16 viral genome was analyzed to better understand the new isolate, and specific regions of the A/AI-1606/16 viral genome were examined (Table 1). The HA protein contains PLRERRRKR↓GLF at the cleavage site, indicating a highly pathogenic phenotype. The virus harbors two conserved amino acid residues in the receptor-binding site of the HA protein (Q226 and G228), suggesting preference for avian-like receptors. Remarkably, the substitutions H107Y, S137A, and T160A were detected in the isolated virus, indicating human-like receptor recognition and the possibility of transmissibility in ferrets [8]. The NA protein contains an 11 amino acid deletion (59–69) in the stalk region, promoting adaptation and enhancing virulence toward poultry and mammals [8]. The NS1 protein contains a five amino acid deletions (80–84), suggesting increased virulence in chickens and mice [27]. Similar to mouse models that have identified substitutions associated with increased viral pathogenicity and replication, substitutions have been detected in the isolated virus in the PB2 protein (L89V, G309D, T339K, R447G, and I495V), M1 protein (N30D and T215A), and NS1 protein (P42S, D92E, and PDZ-motif ESEV) [8]. The molecular markers of NA inhibitors (oseltamivir and zanamivir) [22] and ion-channel inhibitors (amantadine and rimantadine) [26, 28] in the NA and M2 proteins of the A/AI-1606/16 virus exhibited no mutations, suggesting susceptibility to these antiviral influenza drugs.

## Discussion

Clade 2.3.4.4 H5N6 HPAIVs have evolved and been reassorted from Gs/GD since 2013. Based on the HA gene, the clade was clustered into four groups, A, B, C, and D, among which group C is predominant and has been disseminated in Asia and Southeast Asian countries such as China, Vietnam, and Laos [4]. Most prior instances of human H5N6 infections have been from viruses in group C [21]. There are two types of NA genes, reassortant A (no amino acid deletion) and B (an 11 amino acid deletion from 59 to 69 in the stalk region) [29]. Here, in the newly isolated virus, phylogenetic analysis of A/AI-1606/16 revealed that six out of eight genes were most closely related to the H5N6 HPAIV, which has a domestic-poultry-origin in Vietnam between 2015 and 2016. The new isolate also fell into group C and shared over 97% identity with the human isolate A/Guangzhou/39715/2014(H5N6). The NA gene belongs to the reassortant B type. Interestingly, the PB2 and PA genes were closely related to LPAIV strains from domestic ducks in Mongolia (H4N6) and Japan (H3N8), respectively.

The 2.3.4.4 H5N6 HPAI virus was detected in Vietnam immediately after its initial detection in China in 2013 [13]. Nguyen et al. [30] showed that in the period from 2014 to 2017, two predominant clades of H5 HPAI viruses, 2.3.2.1c and 2.3.4.4, evolved from homologous clade viruses endemic to China and prevalent from 2012 to 2013 [5]. Phylogenetic analysis revealed elaborate genetic linkages of the Vietnam and China clade 2.3.4.4 viruses to clade 2.3.2.1c viruses. The recently isolated A/AI-1606/16 virus was detected in chickens in LPMs where many cases of reassorted AIVs were reported [31]. Interestingly, the PB2 and PA genes of A/AI-1606/16 isolate were found to be closely related to LPAIV strains from ducks in Japan (H3N8) and Mongolia (H4N6), which is a long distance away from Vietnam. We thus hypothesized that the parent gene pool of the Japanese and Mongolian LPAIV strains was possibly transmitted to wild birds which may have interacted with poultry before migrating along the East Asian-Australasian flyway to Vietnam [32]. In particular, free-range duck farms where poultry share water sources with wild waterfowl are very popular in Asian countries hence the high risk of AIV reassortment and transmission [33]. Ducks exhibited notably higher resistance to H5N6 HPAIVs compared to chickens [34]. Therefore, we speculated that the newly isolated A/AI-1606/16 virus might have reassorted in free-ranging poultry management practices in Vietnam, at the intersection of wild birds and the poultry sector, and later presented in LPMs.

Vietnam is also part of the East Asia-Australasian flyway and Thinh et al. [35] showed avian influenza A viruses including H5 and H6, and H9 subtypes were isolated from wild migratory birds in Vietnam. Indeed, a live poultry movement network between Vietnam and other neighboring countries revealed the epidemiological links for AIV importation to Vietnam [36]. For example, the clade 7.1 H5N1 HPAIVs were detected in seized chickens at ports of entry in Lang Son Province, and this clade was also detected in an LPM in the Hai Duong Province [37]. Additionally, an A/Muscovy duck/Long An/AI470/2018 (H5N6) isolate in southern Vietnam shared 99% homology with the human isolate A/Guangxi/32797/2018 (H5N6) in China (Fig. 1) [38].

Sites of trade and marketing of live poultries are hot spots for AIV transmission, with multiple subtypes of AIVs, including HPAIVs and LPAIVs, having been detected in LPMs [25]. AIVs have also frequently been detected in poultry farms with poor conditions and those that are free-range [39]. This formed a conducive ecosystem for AIV cohabitation in bird populations. Close contact between humans and live poultries provides opportunities for AIVs to cross the species barrier; indeed, most H7N9 infected human cases have a history of exposure to LPMs [40]. In summary, to control zoonosis of AIVs, enhanced feeding biosecurity, strict transport management, and active surveillance must be implemented.

## Supplementary Information

Below is the link to the electronic supplementary material.

Supplementary file1 Supplementary Figure 1: Identification of the gender of the avian host via CHD and COI gene. (A) CHD gene identification; general banding patterns of 3 primer sets (CHD1F/R, P2/P8, 2550F/2718R) in chicken kidney (control) and #1606 fecal sample for bird sexing (CT=control; F-female; M=male), the female bird contains both W and Z chromosome as the result give to 2 single band (except P2/P8 primer pair give to equal size in the order Galliformes), while the male bird contain only Z chromosome. (B) COI gene identification; bird species identification based on COI gene fragment (approximately 750bp in length). The PCR products were run on a 1.5% agarose gel at 135 volts for 30 mins (DOCX 629 kb)
