# Supplementary material for: Reassortant Highly Pathogenic H5N6 Avian Influenza Virus Containing Low Pathogenic Viral Genes in a Local Live Poultry Market, Vietnam
Source: Curr Microbiol. 2021 Sep 21;78(11):3835–42. doi: 10.1007/s00284-021-02661-z (PMC8486720; doi:10.1007/s00284-021-02661-z)
Supplement: Supplementary file 1 — Supplementary file1 Supplementary Figure 1: Identification of the gender of the avian host via CHD and COI gene. (A) CHD gene identification; general banding patterns of 3 primer sets (CHD1F/R, P2/P8, 2550F/2718R) in chicken kidney (control) and #1606 fecal sample for bird sexing (CT=control; F-female; M=male), the female bird contains both W and Z chromosome as the result give to 2 single band (except P2/P8 primer pair give to equal size in the order Galliformes), while the male bird contain only Z chromosome. (B) COI gene identification; bird species identification based on COI gene fragment (approximately 750bp in length). The PCR products were run on a 1.5% agarose gel at 135 volts for 30 mins (DOCX 629 kb) [file 284_2021_2661_MOESM1_ESM.docx]

**Supplementary figure 1**


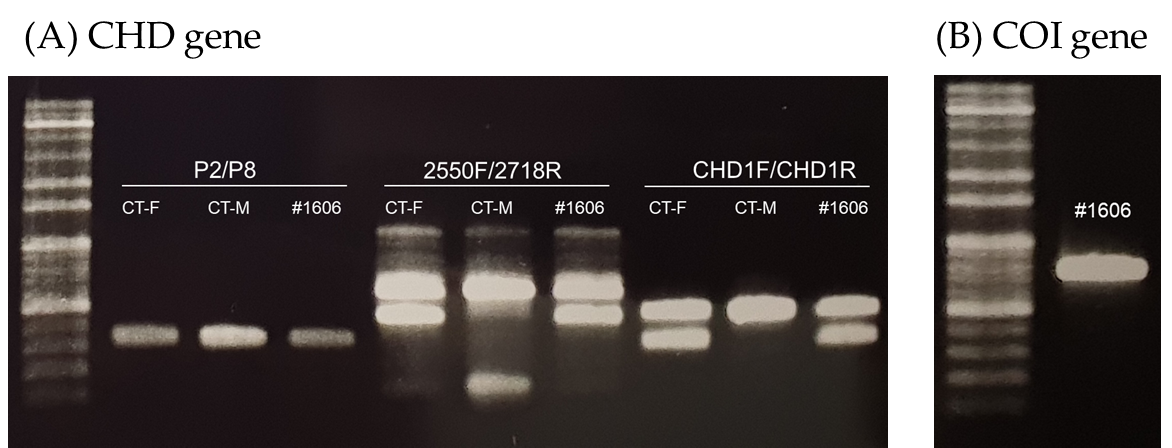


**Supplementary Table 1**. List of fecal, tracheal, and cloacal swab samples from four local live bird markets and poultry farms

| **Collection Date** | **Collection Place** | **Sample Sources** | **No. of Sample** | **AIVs Positive sample** | **Subtype** | **Host** |
| --- | --- | --- | --- | --- | --- | --- |
| 2016-12-30 | Phu Ly Market, Phu Ly City | Market (Fecal) | 35 | 1 | H5N6  (AI-1606/16) | Chicken |
|  | Ngo Khe Commune, Phu Ly City | Farms (Tracheal, Cloacal) | 46 | 0 |  |  |
|  | Yen Nam Commune, Phu Ly City | Farms (Tracheal, Cloacal) | 29 | 2 | H9N2 | Duck |
| 2017-01-06 | Binh My Market, Binh Luc District | Market (Fecal) | 41 | 1 | H5N1 | Unknown |
|  | Binh Nghia Commune, Binh Luc District | Farms (Tracheal, Cloacal) | 26 | 1 | H9N2 | Chicken |
| 2017-01-12 | Vinh Tru Market, Ly Nhan District | Market (Fecal) | 37 | 2 | H5N1 | Unknown |
|  | Nguyen Ly Commune, Ly Nhan District | Farms (Tracheal, Cloacal) | 48 | 0 |  |  |
|  | Hop Ly Commune, Ly Nhan District | Farms (Tracheal, Cloacal) | 63 | 1 | H9N2 | Duck |
| 2017-01-20 | Thi Son Market, Kim Bang District | Market (Fecal) | 28 | 0 |  |  |
| Total | Ha Nam Province | Fecal, Tracheal, Cloacal | 353 | 8 |  |  |
